# Simultaneous Determination of Bergapten, Imperatorin, Notopterol, and Isoimperatorin in Rat Plasma by High Performance Liquid Chromatography with Fluorescence Detection and Its Application to Pharmacokinetic and Excretion Study after Oral Administration of* Notopterygium incisum* Extract

**DOI:** 10.1155/2016/9507246

**Published:** 2016-12-26

**Authors:** John Teye Azietaku, Xie-an Yu, Jin Li, Jia Hao, Jun Cao, Mingrui An, Zhijing Tan, Yan-xu Chang

**Affiliations:** ^1^Tianjin State Key Laboratory of Modern Chinese Medicine, Tianjin University of Traditional Chinese Medicine, Tianjin 300193, China; ^2^College of Material Chemistry and Chemical Engineering, Hangzhou Normal University, Hangzhou 310036, China; ^3^Department of Surgery, University of Michigan, Ann Arbor, MI 48109, USA

## Abstract

A specific, sensitive, and reliable high performance liquid chromatography with fluorescence detection (HPLC-FLD) was first optimized and then used in the simultaneous quantification of bergapten, imperatorin, notopterol, and isoimperatorin in rat plasma using osthole as the internal standard. Liquid-liquid extraction with ethyl acetate was employed in treating the rat plasma samples obtained. Separation was carried out with a Hedera™ ODS column (4.6 × 250 mm, 5 *μ*m) by gradient elution at a temperature of 40°C. Excitation and emission of the fluorescence detector were set to 300 and 490 nm, respectively. The lower limits of quantification for bergapten, imperatorin, notopterol, and isoimperatorin in rat plasma were 4, 40, 4, and 2 ng mL^−1^, respectively. The intraday and interday precision and accuracy for the four coumarins were within acceptable criteria. The recovery of the method was satisfactory with a range of 80.3–114%. The validated method was successfully used for the simultaneous determination of the four coumarins in* Notopterygium incisum* extracts and also for the pharmacokinetic and excretion study of bergapten, imperatorin, notopterol, and isoimperatorin in rats.

## 1. Introduction

In recent years, herbal plants have been extensively studied due to their usage as renewed sources of functional dietary micronutrients and phytochemicals which may be important in treating chronic disorders [1]. Herbal plants serve as alternative source of therapy due to their consistent efficacy levels and affordable prices [2–4]. Herbal plants are also known to contain many compounds that act synergistically to give them their therapeutic effects [5, 6].

*Notopterygium incisum* Ting ex H. T. Chang (family Umbelliferae) is an important traditional Chinese medicinal herb with the rhizomes known as “Qianghuo.” It is well distributed in regions of high altitude in China (i.e., 2500–4000 m), most notable among them being Tibet. The rhizomes and roots of this herb have long been used in treatment of inflammatory diseases such as rheumatoid arthritis, as analgesics are used in the treatment of headache, diaphoretics, and so on [7–10]. “Qianghuo” has been used as part of the ingredient in more than 13 Chinese prescriptions used in the treatment of diseases [11].

Coumarins, essential oils, and phenoloids are among the main chemical constituents found in the roots and rhizomes of* N. incisum* [12–14]. Examples of these include isoimperatorin, notopterol, imperatorin, bergapten, ferulic acid, p-hydroxyphenethyl anisate, and linoleic acid [6, 15]. Many pharmacological researches have been done on* N. incisum* and have reported various therapeutic effects. A lot of research is being carried out on coumarins in recent times due to the enormous pharmacological effects they possess [16]. Studies have indicated that coumarins such as isoimperatorin, notopterol, and bergapten possess anti-inflammatory, analgesic, and anticancer activities [11, 17–19]. These coumarins also have inhibitory effects on 5-lipoxygenase and cyclooxygenase [9, 11, 19] while notopterol's anti-inflammatory effect is as a result of its inhibitory activity on vascular permeability [20]. Bergapten was reported to stimulate melanogenesis in melanoma cells and could serve as anti-gray hair agent [21] and imperatorin has been shown to be active in modulating the gamma-aminobutyric acid (GABA) receptor [22].

Some analytical methods have been developed to simultaneously determine the constituents of* N. incisum* to control its quality. Among these methods are HPLC-UV [23], HPLC-DAD [6], LC-ESI-MS/MS [7], and HPLC-DAD-ESI-MS [9]. Bergapten, imperatorin, notopterol, and isoimperatorin have been used as markers for the fingerprinting analysis and quality control of roots and rhizomes of* N. incisum* [23, 24]. Furthermore, a reverse-phase HPLC was used in quantifying the content of isoimperatorin, bergapten, and notopterol in* N. incisum* and* N. forbesii* to assess the quality of this herb obtained from different regions of China [24]. On the other hand, little pharmacokinetic studies have been reported on this TCM. p-hydroxyphenethyl anisate was used as the only analyte using a RP-LC method [15]. This compound may not be representative of the whole herb since it was the only compound studied among many different compounds present in the herb. Although UPLC–MS/MS was used to determine coumarins in plasma [25], it was expensive for routine analyses. In order to obtain further information on both the pharmacological and clinical effects of* N. incisum*, a pharmacokinetic and quantitative study is required for a relatively cheaper, specific, sensitive, and reliable method for the simultaneous determination of the coumarins which play important role in this TCM.

Fluorescence detection is one of the most sensitive, selective, and specific detection methods for analyzing organic and inorganic compounds. Coumarins are interesting fluorophores with their fluorescence changing drastically depending on the substituents added to the chemical structure and which positions they are introduced in [26]. In this study, we developed a relatively cheaper, specific, sensitive, and reliable HPLC-FLD method for the simultaneous determination of four coumarins in rat plasma and applied this method to the pharmacokinetic and excretion study of* N. incisum* after an oral administration of the extracts to rats.

## 2. Materials and Methods

### 2.1. Plant Material

The rhizome and root of* N. incisum* medicinal herb were purchased from Sichuan province of China. The species were then identified and authenticated by Dr. Lin-Ma (Tianjin University of Traditional Chinese Medicine, Tianjin, China). The voucher specimens were deposited at Tianjin University of Traditional Chinese Medicine.

### 2.2. Chemicals and Reagents

Chengdu Must Biotechnology Co. Ltd. (Chengdu, China) supplied all the reference standards, namely, bergapten, imperatorin, notopterol, isoimperatorin, and internal standard (IS) osthole. Acetonitrile and methanol of HPLC-grade, ethyl acetate, and formic acid were obtained from Tianjin Concord Science Co. Ltd. (Tianjin, China). A Milli-Q Academic system (Millipore, Milford, MA, USA) was used in purifying deionized water used for the HPLC mobile phase.

### 2.3. Apparatus and Chromatographic Conditions

HPLC analysis was performed on an Agilent 1100 HPLC (Agilent Technologies, USA) equipped with a quaternary pump, a degasser, an autosampler, a column thermostat compartment, and a diode array detector (DAD). An Agilent fluorescence detector was coupled to the Agilent system. Separation was carried out with a Hedera ODS column (4.6 mm × 250 mm, 5 *μ*m) by gradient elution at a temperature of 40°C. Excitation and emission of the fluorescence detector were set to 300 nm and 490 nm, respectively. The analysis involved a constant flow rate of 1.0 mL min^−1^ and an injection volume of 30 *μ*L. The mobile phase comprised of (A) aqueous formic acid (0.1%, v/v) and (B) acetonitrile after degassing through ultrasonication using a gradient elution of 15–30% B at 0 to 10 min, 30–42% B at 10 to 20 min, 42% B at 20 to 65 min, 42–80% B at 65–70 min, and 80–90% at 70–75 min. The reequilibration time of gradient elution was 10 min. Owing to the similarity in polarity of imperatorin and notopterol, it was essential to maintain 42% of acetonitrile for 45 min to ensure that the two compounds were well separated.

### 2.4. Preparation of* N. incisum* Extract

1 kg of dried herbs of* N. incisum* was prepared and extracted with 8 L of 90% ethanol for 2 h using reflux extraction method. The ethanol portion was collected and the residue was further extracted under reflux with 8 L of 60% ethanol for 2 h. The solutions were then combined, allowed to cool, and filtered and the extract was concentrated. The extract yield was 20.16%.

### 2.5. Preparation of Stock Solution and Calibration Standards

**Figure 1:**
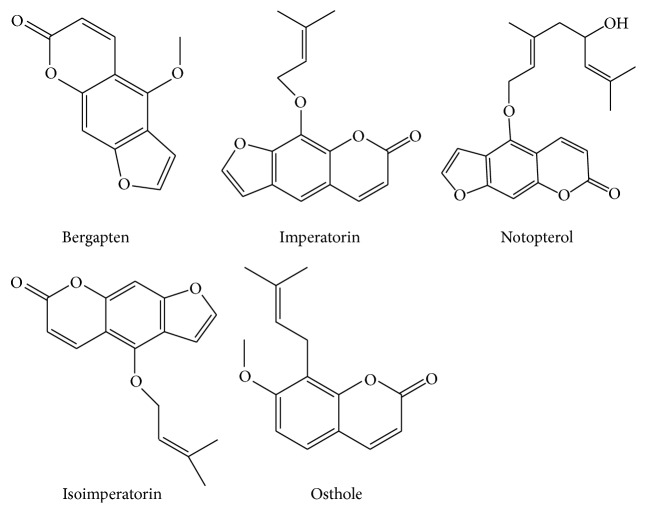
Chemical structures of bergapten, imperatorin, notopterol, osthole (IS), and isoimperatorin.

In preparing the stock solutions, appropriate amounts of bergapten, imperatorin, notopterol, and isoimperatorin were weighed separately and each was dissolved in methanol to achieve a concentration of 1.0 mg mL^−1^. The chemical structures of the four coumarins are shown in Figure 1. The stock solutions of the four analytes were then mixed and then working solutions of mixed standards were prepared by appropriate serial dilution of the mixed stock solution with methanol for use at concentrations of 40–10000 ng mL^−1^ for bergapten, 400–100000 ng mL^−1^ for imperatorin, 40–10000 ng mL^−1^ for notopterol, and 20–5000 ng mL^−1^ for isoimperatorin. The stock solution of IS was also dissolved in methanol and diluted with methanol to a final concentration of 1 *µ*g mL^−1^ and all solutions were stored at 4°C until analysis when they were brought to room temperature before use.

Ten microliter aliquots of the mixed standard of bergapten, imperatorin, notopterol, and isoimperatorin working solutions were added to 100 *μ*L drug-free rat plasma to obtain final concentrations having a range of 4–1000 ng mL^−1^ for bergapten, 40–10000 ng mL^−1^ for imperatorin, 4–1000 ng mL^−1^ for notopterol, and 2–500 ng mL^−1^ for isoimperatorin to prepare the mixed calibration standards.

### 2.6. Sample Pretreatment and Quality Samples

To a 100 *μ*L aliquot of plasma sample, 10 *μ*L IS solution was added. Samples were vortex-mixed for 2 min, extracted with 1000 *μ*L ethyl acetate, and then centrifuged for 10 min at 18000 ×g. Supernatant was transferred into another centrifuge tube and evaporated to dryness using nitrogen gas. The dried residue was reconstituted by adding 100 *μ*L methanol. The solution was shaken and ultrasonicated for 2 min. It was then centrifuged at 18000 ×g for 10 min. A 30 *μ*L of the solution was run with the HPLC and analysis was performed.

Mixed quality control (QC) samples at high, medium, low, and LLOQ concentrations were made by spiking appropriate mixed standard solutions into blank rat plasma, giving plasma concentration ranges of 600, 60, 12, and 4 ng mL^−1^ for bergapten, 6000, 600, 120, and 40 ng mL^−1^ for imperatorin, 600, 60, 12, and 4 ng mL^−1^ for notopterol, and 300, 30, 6, and 2 ng mL^−1^ for isoimperatorin, respectively, followed by the same sample preparation and extraction method described above.

### 2.7. Method Validation

#### 2.7.1. Specificity

Specificity of plasma samples was assessed by comparing blank plasma samples from six different batches, blank plasma samples spiked with 4 compounds, and plasma after oral administration of* N. incisum* extract.

#### 2.7.2. Calibration Curve and LLOQ

Calibration samples were prepared by adding each mixed standard stock solution into blank plasma samples to obtain final concentrations in the ranges of 4–1000 ng mL^−1^ for bergapten, 40–10000 ng mL^−1^ for imperatorin, 4–1000 ng mL^−1^ for notopterol, and 2–500 ng mL^−1^ for isoimperatorin. A plot of the ratio of the peaks area of analytes and internal standard (IS) (*y*) by the corresponding concentration (*x*, ng mL^−1^) was used in obtaining the calibration curve of each compound using a 1/*X* weighted factor. The lower limit of quantification (LLOQ), which is the lowest concentration in the standard curve at which the signal to noise ratio (S/N) was to be larger than 5, with relative standard deviation (RSD, *n* = 6) within 20% and accuracy in the range of 80% to 120% following the USFDA guidelines, was determined.

#### 2.7.3. Precision and Accuracy

Accuracy and precision were done using six batches of mixed QC samples at four levels on the same day (intraday), among three different days (interday), and finally determined using the calibration curve constructed. The intraday and interday precision were stated as the relative standard deviation (RSD), and the accuracy was defined by comparing the measured concentration using the calibration curves to the theoretical concentration of the compounds added to the blank plasma.

#### 2.7.4. Recovery and Stability

The recovery was determined by comparing the observed peak area in extracted plasma samples to those of postprocessed spiked samples. The stabilities of analytes in plasma were assessed by analyzing QC samples (high, medium, low, and LLOQ) under the following conditions: three freeze/thaw cycles, postextraction stability (12 h storage at room temperature), and long-term stability (storage at −80°C for 1 month).

### 2.8. Application to a Pharmacokinetic Study in Rats

The PK study was carried out in accordance with the guidelines for the care and use of laboratory animals and approved by the Animal Ethics Committee of Tianjin University of Traditional Chinese Medicine. Eight male Sprague–Dawley rats weighing 180–220 g were kept at the animal center of Tianjin University of Traditional Chinese Medicine (Tianjin, China) with controlled conditions (temperature at 25 ± 2°C and relative humidity of 55 ± 5%). They were allowed free access to food and water and acclimatized for 7 days until 12 h before the experiment. According to the clinical dose used in human for each day, the rats were administered an oral dose of 4.2 g kg^−1^* N. incisum* extract suspended in 0.5% carboxymethylcellulose sodium salt aqueous solution (CMC-Na) and an amount of blood sample (200 *μ*L) was collected at time 0, 0.083, 0.167, 0.25, 0.5, 0.75, 1, 1.5, 2, 3, 4, 6, 8, 12, and 24 h into heparin-pretreated tubes from the fossa orbitalis of each rat after oral administration of the extract. Blood plasma was immediately obtained by centrifuging the collected blood at 3300 ×g for 10 min and storing at −20°C until analysis.

### 2.9. Excretion Study in Rat Urine, Feces, and Bile

Sixteen male Sprague–Dawley (SD) rats (200 ± 20 g) were divided into two groups (group 1 were for collecting urine and fecal samples in metabolic cages while group 2 were for collecting bile samples from the bile duct using polyethylene tubes). For group 1, the rats were orally given* N. incisum* extract dissolved in 0.5% CMC-Na at a dosage of 4.2 g kg^−1^ and placed in metabolic cages enabling collection of urine and fecal samples separately. The urine and fecal samples were collected at time intervals of 0–4, 4–8, 8–12, 12–24, 24–36, 36–48, 48–60, and 60–72 h. For group 2, the rats were anaesthetized with chloral hydrate at a dose of 0.3 g kg^−1^ administered intraperitoneally. A polyethene tube was used in cannulating the bile duct ensuring continuous flow of bile. The rats were then administered the extract at a dose of 4.2 g kg^−1^ and the bile samples were then collected at different time intervals (0-1, 1-2, 2-3, 3-4, 4-5, 5-6, 6-7, 7-8, and 8-9 h). After the volumes of urine and bile obtained were measured, these samples were stored at −20°C until analysis. The preparation of the urine and bile samples were the same as the plasma sample preparation described above. On the other hand, the fecal samples after collection were dried out in a drying oven at 40°C. After measuring the weights of the fecal samples, they were crushed by a mortar to achieve a uniform powder; 0.1 g of powdered feces was measured and 1 mL methanol was added in 1.5 mL polythene tubes, mixed sufficiently for 3 min, and extracted ultrasonically for 30 min. They were then centrifuged at 18000 ×g for 10 min. Supernatants were transferred into vials for analysis using HPLC-FLD.

### 2.10. Data Analysis

The DAS software (Drug and Statistics 1.0, Medical College of Wannan, China), a computer program, was used in calculating the pharmacokinetic parameters after administering* N. incisum* extract. Bergapten, imperatorin, and isoimperatorin where analyzed using a one-compartment analysis while notopterol was processed using a two-compartment analysis based on Akaike's information criterion (AIC) estimation.

## 3. Result and Discussion

### 3.1. Optimization of the Fluorescence Spectra and Selection of Internal Standard

The excitation and emission wavelengths of the four coumarins were examined to obtain optimized peaks with better signal/noise (S/N). After much testing, maintaining the excitation wavelength and varying the emission wavelength and vice versa, an excitation wavelength of 300 nm and emission wavelength of 490 nm were chosen to be the optimized fluorescence detection wavelength for the simultaneous determination of the four coumarins. An investigation was done into the suitability of the internal standard osthole. It is a furocoumarin and structurally related to the coumarins under study. Furthermore, osthole had a suitable retention time and there was no interference from the endogenous matrix. In addition, the coumarins and osthole were easily separated by HPLC. Although osthole is one of components of* N. incisum* extract in previously published literatures, osthole was not detected in the rat plasmas after oral administration of a dose of 4.2 g kg^−1^* N. incisum* extract. Therefore, osthole was selected as internal standard to determine the four coumarins in* N. incisum*.

### 3.2. Quantification of the Four Coumarins in* N. incisum*

The FLD method was also applied to determine the percentage content of the four coumarins in* N. incisum* extract and, subsequently, the amount of each coumarin given to rats. The contents of bergapten, imperatorin, notopterol, and isoimperatorin are 0.04%, 1.10%, 0.14%, and 0.57%, respectively. The daily dose of* N. incisum* preparation used in clinic is 10 g for an adult, while the real dosage for rat is 4.2 g kg^−1^. Hence, the amount of each coumarin in rat dosage is 1.68 mg kg^−1^ for bergapten, 46.2 mg kg^−1^ for imperatorin, 5.88 mg kg^−1^ for notopterol, and 23.9 mg kg^−1^ for isoimperatorin.

### 3.3. Method Validation

#### 3.3.1. Specificity

**Figure 2:**
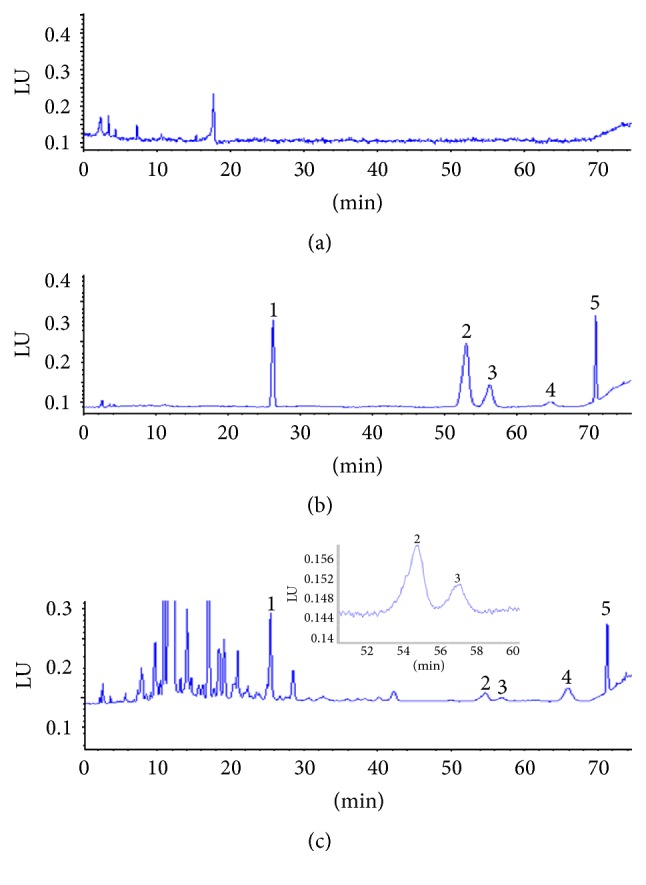
Representative chromatogram of (a) blank rat plasma, (b) blank rat plasma spiked with standard compounds, and (c) real sample after oral administration of* N. incisum*. 1: bergapten, 2: imperatorin, 3: notopterol, 4: osthole, and 5: isoimperatorin.

Figure 2 shows the chromatograms of blank plasma, blank plasma samples spiked with mixed standard compounds, and real plasma sample obtained from a rat following an oral administration of* N. incisum* extract at a dose of 4.2 g kg^−1^. A good resolution was achieved between analytes and IS and no interference from several different sources of rat plasma was observed destructing the separation and quantitation of the four coumarins. In the real pharmacokinetic study samples, no metabolite or endogenous substance interfered with the determination of all analytes.

#### 3.3.2. Calibration Curve and Lower Limits of Quantification

Data on the calibration curves, linearity range, and LLOQ with the regression equations of the four coumarins are provided in Table 1.

#### 3.3.3. Extraction Efficiency

The extraction recoveries of the four coumarins ranged from 80.3% to 114% with their RSDs being less than 15% at all the four mixed concentrations studied (Table 2). The efficiency of extraction was directly dependent on concentration. Hence, the liquid-liquid extraction with ethyl acetate was very efficient in extracting the four coumarins from the plasma sample.

#### 3.3.4. Accuracy and Precision

The intraday and interday accuracy including the precision values are shown in Table 3. The accuracies of the analytes ranged from 86% to 113% while the interday and intraday precision ranged from 3% to 17% for the four coumarins studied. Hence, the reproducibility, accuracy, and reliability of the assay were confirmed.

#### 3.3.5. Stability

The results from the stability tests are shown in Table 4. It was found that, for a period of 24 h, the mixed standard in rat plasma was stable in the autosampler and also after three freeze and thaw cycles. The reduction of the content of the four coumarins in the rat plasma under any of those conditions was not significant after observation. This result demonstrates that the four coumarins could be simultaneously determined in rat plasma using the HPLC method developed.

### 3.4. Pharmacokinetic Studies

**Figure 3:**
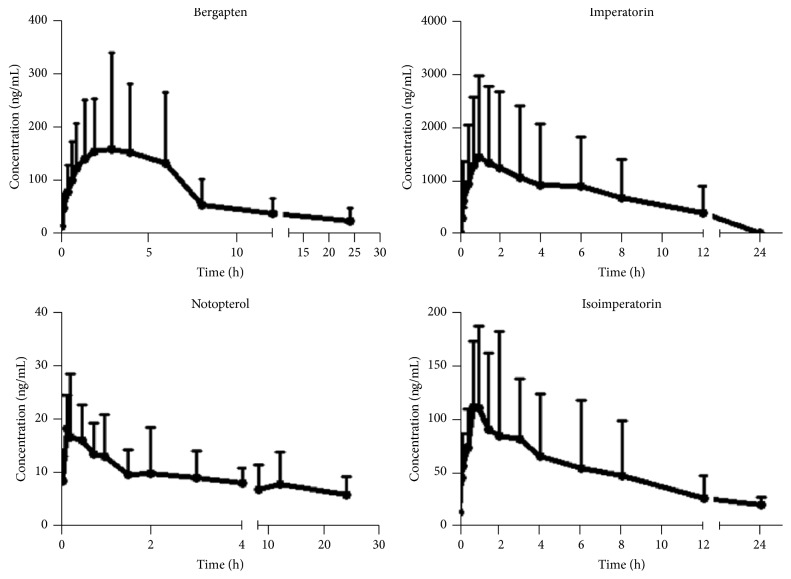
The mean plasma concentration-time profiles of bergapten, imperatorin, notopterol, and isoimperatorin after oral administration of* N. incisum* (*n* = 8, mean ± SD).

The method having been validated was in turn applied to the pharmacokinetic study of the four coumarins after administering a dose of 4.2 g kg^−1^ orally to rats. Pharmacokinetic parameters of the four coumarins were calculated with the aid of a DAS 1.0 pharmacokinetic software with a one-compartment model providing a best fit for the data obtained for the concentration-time curves of the coumarins except for notopterol which behaved as a two-compartment model after oral administration of* N. incisum* extract. The mean concentration-time curves are shown in Figure 3 and the pharmacokinetic parameters are indicated in Table 5. After oral administration of* N. incisum* extract, the absorption (*T*_max_) of notopterol, imperatorin, and isoimperatorin from the rat gastrointestinal tract was discovered to be rapid with all the compounds peaking below 2 h except for bergapten which reached a *T*_max_ at about 3 h. The mean maximum concentrations (*C*_max_) of bergapten, imperatorin, notopterol, and isoimperatorin in rat plasma were 183.5 ± 171.4, 1667 ± 1504, 23.31 ± 6.87, and 145.1 ± 75.9 ng mL^−1^, respectively. Coumarins are important plant constituents known to have various pharmacological effects. From the results, most of the coumarins exhibited a rapid absorption into the blood of the rats with notopterol having the fastest time for it to be absorbed completely into the blood depending on the *T*_max_. Bergapten exhibited the long elimination half-life of 12 h which implies that it takes a longer time for bergapten to be cleared in rat tissues. Bergapten could be strongly protein bound in blood accounting for this observation. Further investigations need to be done to elucidate the reason for this. On the other hand, imperatorin, notopterol, and isoimperatorin exhibited a shorter half-life within 6 h; hence these compounds were eliminated faster in the blood when compared with bergapten. The half-life of imperatorin was 4.99 ± 3.02 h which was similar to that observed in a previous report [26]. The total drug exposure in rats in over 24 h, AUC_0–24_, is 1638 ± 2490, 10975 ± 11955, 101.6 ± 34.8, and 830.5 ± 762.4 ng mL^−1^ h for bergapten, imperatorin, notopterol, and isoimperatorin, respectively.

### 3.5. Excretion Study of Analytes in Rat Urine, Feces, and Bile

**Figure 4:**
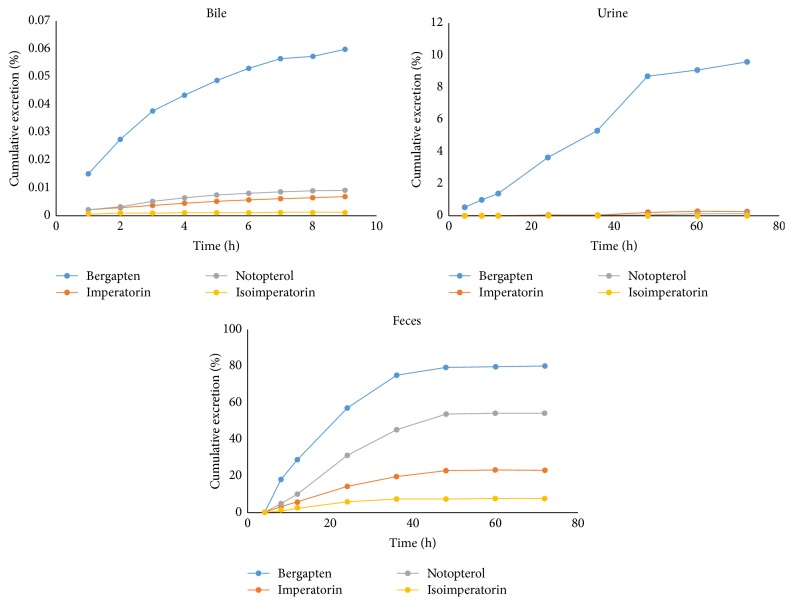
Bile, urinary, and fecal cumulative excretion of the four analytes in rats following oral administration at a dose of 4.2 g kg^−1^* N. incisum* extract.

The cumulative excretion of bergapten, imperatorin, notopterol, and isoimperatorin in urine, feces, and bile was determined as shown in Figure 4. After an oral administration of* N. incisum* extract at a dose of 4.2 g kg^−1^ to the rats, all the coumarins could be detected in rat urine until 72 h. The four coumarins increased in amount in the bile gradually until about 6 h and remained stable for the rest of the time except for imperatorin and isoimperatorin that gave a gradual increase throughout the whole time of excretion in the bile.

In urine, bergapten levels were observed to have increased quite rapidly from 12 to 48 h. The cumulative excretion of bergapten in urine was 9.5724 + 1.6891%. Those for imperatorin, notopterol, and isoimperatorin were 0.2759 + 0.0493%, 0.1171 + 0.0096%, and 0.0084 + 0.0016%, respectively. Imperatorin and notopterol levels increased rapidly from 36 to 48 h and then plateaued to 72 h. In the fecal sample, bergapten exhibited the highest cumulative excretion with about 80% of bergapten excreted unchanged in fecal sample. The levels of all the compounds increased rapidly from 12 h to 48 h and then tapered to 72 h. The cumulative excretions are 23.4595 + 2.2545%, 54.1083 + 4.4359%, and 7.7284 + 2.2114% for imperatorin, notopterol, and isoimperatorin, respectively. The results suggested that these compounds were excreted greatly through the fecal route.

## 4. Conclusion

A specific, sensitive, and reproducible HPLC-FLD method was newly developed for the simultaneous determination of bergapten, imperatorin, notopterol, and isoimperatorin and this was in turn applied to the pharmacokinetic and excretion study of bergapten, imperatorin, notopterol, and isoimperatorin in rats after oral administration of* N. incisum* extracts. This method was accurate and precise using a simple liquid-liquid extraction to treat the rat plasma. Pharmacokinetic and excretion study of the four coumarins (bergapten, imperatorin, notopterol, and isoimperatorin) can be applied in evaluating the clinical efficiency of* N. incisum*, looking at the drug interactions and how these compounds elicit their synergistic effect.

## Figures and Tables

**Table 1:** Calibration curve and LLOQ.

Compounds	Regression equation	*r* ^2^	Linearity range (ng mL^−1^)	LLOQ (ng mL^−1^)
Bergapten	*Y* = 0.0129*X* − 0.0091	0.997	4–1000	4
Imperatorin	*Y* = 0.0020*X* + 0.0513	0.994	40–10000	40
Notopterol	*Y* = 0.0136*X* − 0.0492	0.997	4–1000	4
Isoimperatorin	*Y* = 0.019*X* + 0.0042	0.997	2–500	2

**Table 2:** Extraction recovery of four coumarins in rat plasma (*n* = 6).

Compounds	Concentration (ng mL^−1^)	Recovery	
		Mean (%)	RSD (%)
Bergapten	4	87.7	3.7
	12	94.9	14.7
	60	112	6.67
	600	105	4.63

Imperatorin	40	112	3.58
	120	96.7	11.9
	600	114	3.83
	6000	101	3.71

Notopterol	4	80.3	9.48
	12	112	9.15
	60	98.5	6.51
	600	103	4.82

Isoimperatorin	2	105	12.3
	6	98.2	11.9
	30	101	7.94
	300	99.5	8.64

**Table 3:** Intraday, interday accuracy, and precision (*n* = 6).

Compounds	Concentration	Interday		Intraday	
	(ng mL^−1^)	Mean (%)	RSD (%)	Mean (%)	RSD (%)
Bergapten	4	102	9.43	102	9.80
	12	98.0	8.26	91.1	11.2
	60	86.4	3.69	86.1	6.28
	600	94.9	3.27	89.2	8.01

Imperatorin	40	94.9	17.3	99.3	13.6
	120	102	9.61	94.3	8.48
	600	94.2	6.80	92.1	7.78
	6000	100	6.72	97.3	5.69

Notopterol	4	103	11.5	101	12.3
	12	114	7.89	97.6	11.8
	60	88.9	4.69	86.2	13.6
	600	88.9	9.44	95.1	9.67

Isoimperatorin	2	93.7	14.2	97.0	13.5
	6	86.7	3.52	94.0	11.9
	30	107	10.5	94.0	10.7
	300	113	3.55	96.9	11.9

**Table 4:** Stability of bergapten, imperatorin, notopterol, and isoimperatorin in rat plasma (*n* = 6).

Compounds	Concentration	Freeze-thaw cycles		Autosampler for 24 h		At −80°C for 1 month	
	(ng mL^−1^)	Mean (%)	RSD (%)	Mean (%)	RSD (%)	Mean (%)	RSD (%)
Bergapten	4	106	11.1	100	10.2	80.5	4.65
	12	91.1	6.09	101	12.9	91.1	9.57
	60	83.6	7.69	115	6.93	104	6.40
	600	85.1	5.83	88.9	6.99	112	4.05

Imperatorin	40	93.7	14.7	105	8.13	113	6.86
	120	93.9	4.74	115	4.21	85.7	7.24
	600	106	4.02	89.6	3.92	98.3	8.85
	6000	100	4.65	114	1.85	104	4.78

Notopterol	4	102	14.7	104	12.1	92.3	12.5
	12	93.2	10.1	109	13.6	88.6	8.38
	60	97.1	9.76	97.3	9.39	105	9.86
	600	94.9	2.39	102	5.61	103	5.18

Isoimperatorin	2	93.9	7.40	96.9	12.2	82.2	15.0
	6	106	9.71	119	10.0	112	9.12
	30	85.7	7.80	98.1	9.41	111	7.96
	300	85.8	5.80	88.8	7.34	103	5.72

**Table 5:** Pharmacokinetic parameters of four coumarins after oral administration of *N. incisum* extract (*n* = 8, mean ± SD).

Parameters	Bergapten	Imperatorin	Notopterol	Isoimperatorin
*C*_max_ (ng mL^−1^)	183.5 ± 171.4	1667 ± 1505	23.31 ± 6.87	145.1 ± 75.9
*T*_max_ (h)	3.06 ± 3.71	1.65 ± 1.80	0.45 ± 0.35	1.59 ± 0.97
*T*_1/2_ (h)	12.8 ± 22.2	4.99 ± 3.02	3.18 ± 3.44	5.62 ± 4.06
AUC_0–24_ (ng/mL h)	1638 ± 2490	10975 ± 11955	101.6 ± 34.8	830.5 ± 762.4
AUC_0–*∞*_ (ng/mL h)	1958 ± 2490	13948 ± 13926	296.2 ± 241.8	2123 ± 2866

## References

[1] Ozcan T., Akpinar-Bayizit A., Yilmaz-Ersan L., Delikanli B. (2014). Phenolics in human health. *International Journal of Chemical Engineering and Applications*.

[2] Efferth T. (2011). Perspectives for globalized natural medicines. *Chinese Journal of Natural Medicines*.

[3] Guan T.-Y., Liang Y., Li C.-Z., Xie L., Wang G.-J., Sheng L.-S. (2011). Recent development in liquid chromatography/mass spectrometry and allied topics for traditional Chinese medicine research. *Chinese Journal of Natural Medicines*.

[4] Hu C., Xu G. (2014). Metabolomics and traditional Chinese medicine. *TrAC—Trends in Analytical Chemistry*.

[5] Gu M., Ouyang F., Su Z. (2004). Comparison of high-speed counter-current chromatography and high-performance liquid chromatography on fingerprinting of Chinese traditional medicine. *Journal of Chromatography A*.

[6] Jiang F., Tao Y., Shao Y. (2007). Fingerprinting quality control of Qianghuo by high-performance liquid chromatography-photodiode array detection. *Journal of Ethnopharmacology*.

[7] Li Y.-H., Jiang S.-Y., Guan Y.-L. (2006). Quantitative determination of the chemical profile of the plant material ‘Qiang-huo’ by LC-ESI-MS-MS. *Chromatographia*.

[8] Shi J., Jiang S.-Y., Ma X.-J., Sun H., Zhou Y. (2007). Studies on seeds germination and seedlings growth of *Notopterygium incisum*. *China Journal of Chinese Materia Medica*.

[9] Xu K., Jiang S., Zhou Y. (2011). Discrimination of the seeds of *Notopterygium incisum* and *Notopterygium franchetii* by validated HPLC-DAD-ESI-MS method and principal component analysis. *Journal of Pharmaceutical and Biomedical Analysis*.

[10] (2010). *Commission CP: Pharmacopoeia of the People's Republic of China*.

[11] Tang S. Y., Wang H., Zhang W., Halliwell B. (2008). *Notopterygium forbesii* Boiss extract and its active constituents increase reactive species and heme oxygenase-1 in human fetal hepatocytes: mechanisms of action. *Chemical Research in Toxicology*.

[12] Wu S. B., Yu Y. H., Hu Y. H., Hu J. F. (2008). A new dimeric furanocoumarin from *Notopterygium incisum*. *Chinese Chemical Letters*.

[13] Xiao Y. Q., Sun Y. F., Liu X. H. (1994). Chemical constituents of *Notopterygium incisum* Ting. *China Journal of Chinese Materia Medica*.

[14] Zhang P., Yang X.-W. (2008). Studies on chemical constituents in roots and rhizomes of *Notopterygium incisum*. *China Journal of Chinese Materia Medica*.

[15] Zhang Y.-B., Yang X.-W. (2009). Rapid and sensitive RP-LC method for the quantification and pharmacokinetic study of p-hydroxyphenethyl anisate in rat plasma. *Chromatographia*.

[16] Chang Y.-X., Zhang Q.-H., Li J. (2013). Simultaneous determination of scopoletin, psoralen, bergapten, xanthotoxin, columbianetin acetate, imperatorin, osthole and isoimperatorin in rat plasma by LC-MS/MS for pharmacokinetic studies following oral administration of Radix Angelicae Pubescentis extract. *Journal of Pharmaceutical and Biomedical Analysis*.

[17] Guo Y. H., Sha M., Meng X. S., Cao A. M. (2005). The Anti-viral Studies of Notopterygium incisum. *Lishizhen Medicine and Materia Medica Research*.

[18] Santana L., Uriarte E., Roleira F., Milhazes N., Borges F. (2004). Furocoumarins in medicinal chemistry. Synthesis, natural occurrence and biological activity. *Current Medicinal Chemistry*.

[19] Wu S.-B., Pang F., Wen Y., Zhang H.-F., Zhao Z., Hu J.-F. (2010). Antiproliferative and apoptotic activities of linear furocoumarins from *Notopterygium incisum* on cancer cell lines. *Planta Medica*.

[20] Okuyama E., Nishimura S., Ohmori S., Ozaki Y., Satake M., Yamazaki M. (1993). Analgesic component of *Notopterygium incisum* Ting. *Chemical and Pharmaceutical Bulletin*.

[21] Matsuda H., Hirata N., Kawaguchi Y. (2005). Melanogenesis stimulation in murine B16 melanoma cells by umberiferae plant extracts and their coumarin constituents. *Biological and Pharmaceutical Bulletin*.

[22] Zaugg J., Eickmeier E., Rueda D. C., Hering S., Hamburger M. (2011). HPLC-based activity profiling of *Angelica pubescens* roots for new positive GABAA receptor modulators in *Xenopus* oocytes. *Fitoterapia*.

[23] Kim S., Kim K. Y., Han C. S. (2012). Simultaneous analysis of six major compounds in osterici radix and Notopterygii rhizoma et radix by HPLC and discrimination of their origins from chemical fingerprint analysis. *Archives of Pharmacal Research*.

[24] Qian G.-S., Wang Q., Leung K. S.-Y., Qin Y., Zhao Z., Jiang Z.-H. (2007). Quality assessment of Rhizoma et Radix Notopterygii by HPTLC and HPLC fingerprinting and HPLC quantitative analysis. *Journal of Pharmaceutical and Biomedical Analysis*.

[25] Wu Y., Wang F., Ai Y. (2015). Simultaneous determination of seven coumarins by UPLC-MS/MS: application to a comparative pharmacokinetic study in normal and arthritic rats after oral administration of Huo Luo Xiao Ling Dan or single-herb extract. *Journal of Chromatography B*.

[26] Murata C., Masuda T., Kamochi Y. (2005). Improvement of fluorescence characteristics of coumarins: syntheses and fluorescence properties of 6-methoxycoumarin and benzocoumarin derivatives as novel fluorophores emitting in the longer wavelength region and their application to analytical reagents. *Chemical & Pharmaceutical Bulletin*.

